# Living Young With Lupus: A Case Report on the Challenges of Multi-System Disease in a Young Adult

**DOI:** 10.7759/cureus.96946

**Published:** 2025-11-16

**Authors:** Pavraj Manu, Kavitha Nadesalingam

**Affiliations:** 1 Rheumatology, Bradford Royal Infirmary, Bradford, GBR

**Keywords:** juvenile-onset, lupus nephritis, medico-legal issues, pericardial effusion, systemic lupus erythematosus, transitional care, treatment non-adherence

## Abstract

Systemic erythematosus lupus (SLE) is a multisystem autoimmune disease that can present aggressively within young people. The presence of severe multi-organ disease poses multiple challenges including physical, psycho-social, and, in this case, medico-legal considerations. We present the case of a young person’s journey in navigating these complexities. A 16-year-old girl, whilst on holiday abroad, presented with polyarthralgia, weight loss, severe fatigue and lower limb myalgia. Immunological markers revealed high titres of antinuclear antibody (ANA), anti-double-stranded DNA (anti-dsDNA) and anti-Smith (anti-Sm) antibodies, confirming a diagnosis of SLE. On return to the United Kingdom (UK), she developed recurrent pericardial effusion, pleural effusion and suspected lupus nephritis ensued after difficulties with treatment adherence and declining of renal biopsy. This case explores how personal autonomy, psychosocial health and treatment adherence intersect to impact disease progression and patient outcome. Ultimately, this case highlights the importance of utilising a cross-specialty approach and seeking governance support early for preparedness in acute situations and support complex decision-making. This must be balanced with careful planning to empower young people with SLE to take an active role in their condition, which can be achieved with utilisation of rheumatology transition clinics.

## Introduction

Systemic lupus erythematosus (SLE) is a multisystem autoimmune disease characterised by loss of immune self-tolerance, autoantibody production, immune complex deposition, and complement consumption, which leads to inflammation and tissue injury [1]. Classification is based on clinical and serological criteria established by the European Alliance of Associations for Rheumatology (EULAR) and the American College of Rheumatology (ACR). Antinuclear antibody (ANA) positivity is the main criterion, and subsequent weighted items, some of which include mucocutaneous, musculoskeletal, renal, haematological, and serological domains [1].

Juvenile-onset systemic lupus erythematosus (jSLE) is defined as disease onset before 18 years old and accounts for roughly 15-20% patients [2]. The literature suggests that early onset typically exhibits higher disease activity than adult onset and increased likelihood of major-organ involvement (notably renal, cardiopulmonary, and haematological). Subsequently, this higher disease activity leads to greater long-term morbidity and mortality. This often requires the use of more aggressive immunosuppressive therapy, which brings wider implications such as exposure to a range of side effects at a young age [2,3].

Clinical management is further complicated, particularly within jSLE, as only 50-60% of patients take their medication as prescribed [4]. The reasons for treatment hesitancy are complex, with current research pointing towards multifactorial factors which ultimately reflect a divide between patient and caregiver values that centre around developmental and psychosocial concerns. Studies show that the common themes among patients include restrictions on life decisions, prognostic uncertainty and resentment of long-term treatment [5]. In contrast, caregivers are frequently concerned regarding disruption of family schedule, schooling and maintaining a sense of normalcy for their child [4].

Furthermore, the progression into adolescence marks a transition from paediatric to adult care. This transition is associated with care fragmentation, with contributing factors such as shifting roles for caregivers and clinicians, evolving capacity in shared decision making and brings a higher risk of disengagement from care. Common barriers often include limited access to non-standardised transition care services, limited readiness, unmet mental-health needs and differing ideas about autonomy [6].

Within the United Kingdom (UK), consent, capacity, and best-interest considerations add further complexity when serious disease coexists with treatment refusal, requiring careful navigation of ethical and medico-legal frameworks in partnership with young adults and their caregivers.

This case presents a 16-year-old girl with jSLE to: (i) highlight how treatment hesitancy during the transition period can precipitate multi-organ involvement and (ii) discuss the real-world challenges in managing a 16-year-old girl with jSLE and the UK medico-legal considerations that arise when treatment is declined.

## Case presentation

A 15-year-old girl, originally from Eastern Europe, was admitted under paediatric rheumatology while visiting family abroad. She initially presented with a three-week history of polyarthralgia, severe fatigue and lower limb myalgia. This was followed by a one-week history of oral ulcers and lower limb rash resembling livedo reticularis. Over this four-week period, she also had a clinically significant weight loss of 4 kg, with her baseline weight equating to 44 kg. She was diagnosed with jSLE with positive ANA, anti-double-stranded DNA (anti-dsDNA) and anti-Smith antibodies (anti-Sm). The patient had no relevant past medical history, drug history or family history.

Further investigations abroad revealed multi-organ involvement, including abnormal liver function tests with hepatomegaly on ultrasound, suggesting lupus hepatitis; haematological involvement (anaemia and leucopoenia) and lupus nephritis based on clinical presentation and results from non-invasive investigations. Exact serology results with reference ranges were not available on the discharge letter from the hospital abroad; therefore, investigations had to be restarted on presentation to the UK.

The patient was discharged on hydroxychloroquine; however, after returning to the UK, she self-discontinued treatment, which resulted in disease progression. Subsequently, she was referred to a district general hospital and seen within adult rheumatology services as she had turned 16 years old. Although treatment hesitancy persisted, it improved with appropriate counselling with a multidisciplinary approach; however, treatment was still not optimised.

The following section discusses the challenges that arose when establishing the base of immunosuppressive medication for her jSLE. The patient demonstrated good compliance with hydroxychloroquine throughout her treatment. She also demonstrated good compliance with prednisolone, which was prescribed for each flaring episode of her jSLE, with the dose starting between 0.5mg and 1mg/kg/day, dependent on the severity of her flare and weaned down accordingly. A further immunosuppressant was agreed upon to control her flares and minimise steroid therapy. The patient struggled with the addition of further immunosuppressive therapy in the form of mycophenolate mofetil due to intolerable gastrointestinal side effects, including abdominal pain and nausea. Mycophenolate mofetil was discontinued and replaced with enteric-coated mycophenolate sodium, which was selected to improve gastrointestinal tolerability while maintaining equivalent mycophenolic acid exposure. The following part of the case study does not follow the clinical course in a chronological order but focuses on three key disease complications and associated important themes, which were all intertwined.

Lupus nephritis and treatment hesitancy

**Figure 1:**
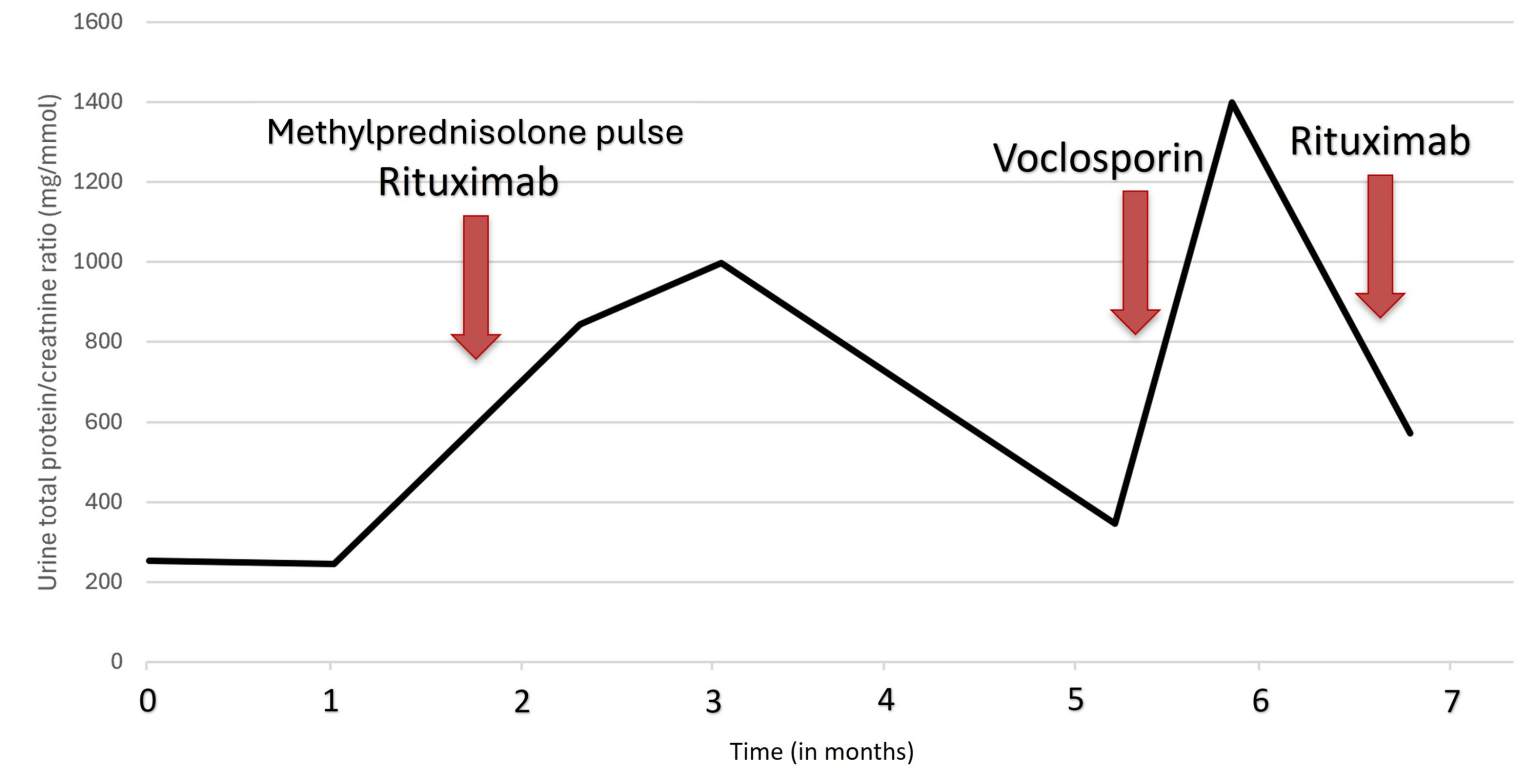
Serial measurements of urine protein-to-creatinine ratio (uPCR) over time in a patient with assumed lupus nephritis. The Y axis represents the uPCR measured in mg/mmol. The X axis represents time in months since the patient first presented to a UK rheumatology department. The first arrow (leftmost) indicates the initiation of IV methylprednisolone (500 mg) pulse therapy and rituximab (1 g). The second arrow (middle) indicates the initiation of voclosporin (23.7 mg twice daily). The third arrow indicates the second cycle of rituximab (1 g).

Concern was raised when persistent haemato-proteinuria was noted on urinalysis alongside a rapidly deteriorating urine protein-to-creatinine ratio (uPCR) as seen in Figure 1, continuing until the second month since arriving back in the UK. Despite repeated counselling and early involvement of the renal physicians, the patient continued to decline a renal biopsy. This refusal was due to concerns regarding some of the previously counselled risks of biopsy, including bleeding, pain and infection. The patient understood the importance of how histology can alter management and declined again, even with the offer of a general anaesthetic. In the absence of a renal biopsy, plans were advanced in favour of treating this as likely lupus nephritis given the clinical picture. After discussion with a local specialist lupus centre, rituximab was approved for the treatment of lupus nephritis. However, the patient initially refused this escalation of treatment, considering it “too aggressive” despite appropriate counselling.

This medication hesitancy, combined with biopsy refusal described above, further added to the challenges of clinical management. Guidance from the paediatric team led to signposting to young lupus resources and an attempt to meet with another young adult with lupus to talk about shared experiences. Fortunately, this led to the patient agreeing to the initiation of rituximab and methylprednisolone pulse therapy, as seen in the initial arrow on Figure 1 around month two. Although the uPCR continued to climb until month three, it eventually showed a steady decline until month five. This demonstrated a delay of almost a return to baseline in uPCR response to rituximab of three months, which is roughly the time stated in the literature for rituximab to exert its effect [7]. 

The uPCR began to rapidly deteriorate once again just after the fifth month, as seen in Figure 1. After initially refusing voclosporin, the patient agreed once approval was again sought from the local lupus specialist centre. This resulted in a marked improvement by the seventh month, which supports the median time (roughly 29 days) in the literature for voclosporin to significantly improve uPCR [8]. The patient tolerated voclosporin for five weeks before discontinuing due to gastrointestinal side effects.

Pleural effusion and refusal of treatment

**Figure 2:**
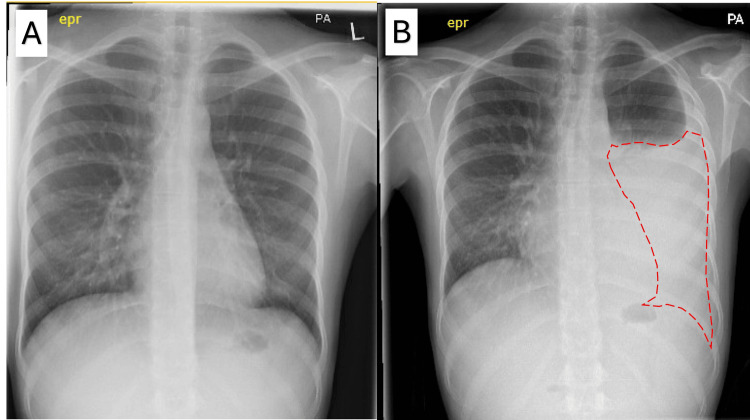
(A) Posterior-anterior chest radiograph showing baseline appearance before disease activity (left) and (B) large volume left-sided pleural effusion six months later as illustrated by the red dotted line (right).

**Figure 3:**
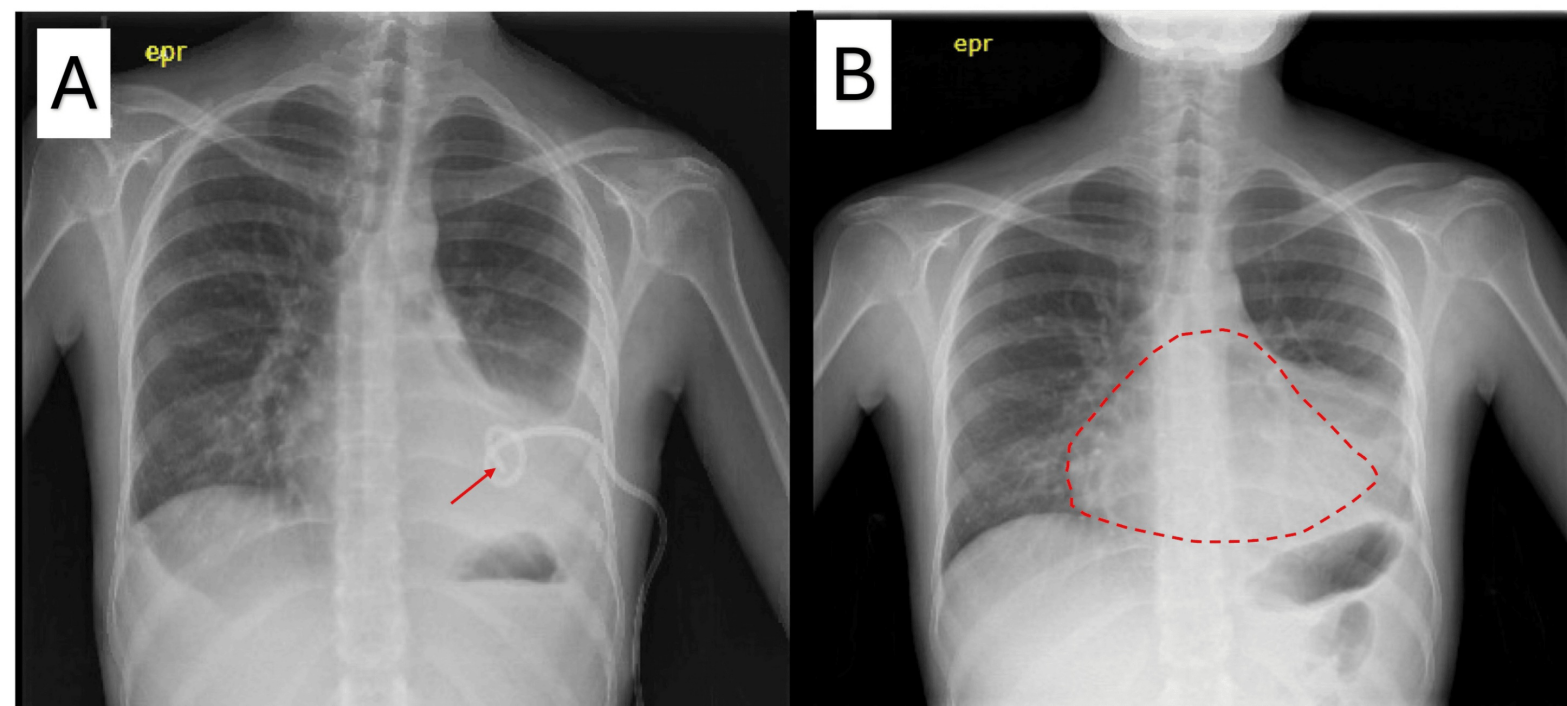
(A) Posterior-anterior chest radiograph showing partial resolution of left-sided pleural effusion with chest drain insertion. The red arrow points towards the correctly sited chest tube (left) and (B) repeat chest radiograph two months later prompting concern for a pericardial effusion with the red dotted line highlighting cardiomegaly (right).

A symptomatic large volume left-sided pleural effusion was discovered on a chest radiograph (Figure 2A) after prematurely stopping a weaning course of prednisolone and non-compliance with mycophenolate. The respiratory physicians planned admission for insertion of a chest drain, but both the patient (aged 16 years at the time and considered a minor under UK law) and her parent initially were hesitant and declined admission. The legal team was consulted, as the patient was deemed to be competent, and there was concern that she and her parent would refuse lifesaving care. In this scenario, although rare, the courts can overturn the young person’s refusal of life-sustaining treatment, and the hospital team was prepared should this situation arise. Fortunately, it did not come to this, and the patient agreed to admission and had a chest drain inserted (Figure 3A). After inadequate drainage, the patient was referred to cardiothoracic surgery, who performed video-assisted thoracoscopic surgery to resolve the effusion.

Pericardial effusion and attempt to fly

A follow-up chest X-ray showed a reduction in the size of the pleural effusion; however, it also raised concern for a pericardial effusion (Figure 3B). Echocardiogram revealed a moderate-sized global effusion with no valve abnormalities and preserved systolic function (left ventricular ejection fraction = 60-65%). This occurred in the context of serologically active SLE, with elevated anti-dsDNA (300 IU/mL; normal 0-9.99 IU/mL), positive anti-Sm (>8 IU/mL; normal 0-0.99 IU/mL), hypocomplementemia (C3 0.71 g/L; normal 0.75-1.60 g/L, C4 0.02 g/L; normal 0.20-0.65 g/L), and an elevated ESR (34 mm/hr; normal 0-12 mm/hr).

A joint decision among cardiology, rheumatology and renal was made to escalate treatment to rituximab and methylprednisolone pulse therapy, which was approved by the local specialist lupus centre. This was followed by further treatment hesitancy and refusal.

Matters were complicated further when the patient and her mother arranged to return to their native country against medical advice. This was to visit family to help lift the patient’s mood. It was emphasised that this was against medical advice and the G force of flying could lead to haemodynamic instability in a pre-existing pericardial effusion (leading to a cardiac tamponade) [9]. Fortunately, the patient decided against travel after repeated counselling about the dangers of travel while her lupus was active and evolving.

The patient was again counselled on the importance of medication adherence, particularly given ongoing concerns about side effects, and psychological support was offered. This was supported by historical data that showed that the five-year survival rate for untreated SLE was approximately 50% in the 1950s, improving to over 90% since the 1990s with advances in therapy [10]. The patient agreed to pulse therapy with methylprednisolone but again declined rituximab. On a repeat echocardiogram two weeks later, the pericardial effusion had resolved. Four months later, this effusion had returned, which led to the starting of rituximab with further pulse therapy (this was the same rituximab initiation for lupus nephritis discussed above). The effusion had resolved on a repeat echocardiogram five months later.

## Discussion

This case explored some of the known challenges in managing SLE, particularly juvenile-onset disease. Treatment refusal in this case mirrored reasons shared amongst people living with jSLE, including adverse effects of medication, perceived ‘aggressiveness’ of treatment, evolving autonomy (desire to travel) and transition in care.

Addressing these challenges requires an intricate and stepwise approach such as adoption of transitional care. Transitional care is the "planned movement of adolescents with chronic conditions from paediatric to adult healthcare" [11]. Depending on the hospital, this service typically follows a phased model from ages 10 to 25 years, encompassing adolescent, teenage, and young adult clinics before transfer to adult services. The age at which a patient progresses through the clinics is based on rough age criteria and assessment of the readiness of the individual patient.

At the treating hospital, a structured transition clinic supports patients moving from paediatric to adult services. Patients aged 16 years and older are typically transferred directly to adult care; therefore, this patient entered adult services without following the transition pathway. This hospital houses its own paediatric rheumatology service, an uncommon feature outside tertiary centres, which allows for continuity of specialist care but currently does not have an intermediate young adult service. Stepwise transition models, where available, have been shown to promote autonomy, confidence, and engagement in self-management, contributing to improved clinical and psychosocial outcomes [12].

As the patient was under 18 years of age and refused treatment, this case was challenging from a medico-legal perspective. Considerations were made of the legal implications both when she refused treatment for her large pleural effusion and when she wished to fly against medical advice with a significant pericardial effusion.

This highlighted the importance of seeking both local legal support and General Medical Council guidance on consent and decision making in young adults [13]. This applied as both the patient and her mother refused treatment that was considered to be life-sustaining and in the best interests of the patient. There was emphasis from the legal team on clear documentation of counselling, capacity (both parent and patient) and escalation when there were concerns for refusal of lifesaving/protecting treatment in managing the pleural and pericardial effusions. This preparedness was crucial if the Court of Protection were to be considered in an acute scenario. The Court of Protection is a specialist mental capacity court in England and Wales created by the Mental Capacity Act in 2005. It has the authority to make a decision on behalf of the subject in their best interests if capacity is lacking [14]. This pathway can be sought if the patient is aged 16 or 17 and declines treatment alongside their parent, which may lead to death or permanent injury [15].

## Conclusions

On seeking patient consent to write up this case report, the patient herself wanted to educate care givers on how managing a young adult requires additional considerations specifically around coming to terms with having a long-term condition. The patient wanted health care professionals to understand that treating a teenager is different to treating an older adult and how it felt unfair to her that she was having to live with a long-term condition while her peers were well and led a "normal life". Access to clinical psychology services, which this patient has since utilised, can be beneficial in helping patients accept their diagnosis and overcome treatment hesitancy. We would recommend all departments managing young adults with long term conditions have access to clinical psychology although due to resources, access varies across the country and between different specialties.

A significant additional consideration in this case was the patient’s age and concerns that she would refuse lifesaving treatment. Seeking timely medico-legal advice in these situations is necessary to facilitate early escalation and planning. A delicate balance should ultimately be sought between safeguarding a young person’s welfare and respecting their developing independence. This further supports the importance of transition care, which aims to align best interests with personal autonomy through structured support and collaborative decision-making.

## References

[1] Aringer M, Costenbader K, Daikh D (2019). 2019 European League Against Rheumatism/American College of Rheumatology Classification Criteria for Systemic Lupus Erythematosus. Arthritis Rheumatol.

[2] Huerta-Calpe S, Del Castillo-Velilla I, Felipe-Villalobos A, Jordan I, Hernández-Platero L (2023). Severe juvenile-onset systemic lupus erythematosus: a case series-based review and update. Children (Basel).

[3] Charras A, Smith E, Hedrich CM (2021). Systemic lupus erythematosus in children and young people. Curr Rheumatol Rep.

[4] Harry O, Crosby LE, Smith AW (2019). Self-management and adherence in childhood-onset systemic lupus erythematosus: what are we missing?. Lupus.

[5] Tunnicliffe DJ, Singh-Grewal D, Chaitow J (2016). Lupus means sacrifices: perspectives of adolescents and young adults with systemic lupus erythematosus. Arthritis Care Res (Hoboken).

[6] Colver A, Rapley T, Parr JR (2019). Facilitating the transition of young people with long-term conditions through health services from childhood to adulthood: the Transition research programme. Program Grants Appl Res.

[7] Rovin BH, Furie R, Latinis K (2012). Efficacy and safety of rituximab in patients with active proliferative lupus nephritis: the Lupus Nephritis Assessment with Rituximab study. Arthritis Rheum.

[8] Rovin BH, Teng YKO, Ginzler EM (2021). Efficacy and safety of voclosporin versus placebo for lupus nephritis (AURORA 1): a double-blind, randomised, multicentre, placebo-controlled, phase 3 trial. Lancet.

[9] D'Arcy JL, Manen O, Davenport ED (2019). Heart muscle disease management in aircrew. Heart.

[10] Lao C, White D, Rabindranath K, Van Dantzig P, Foxall D, Lawrenson R (2024). Mortality and causes of death in systemic lupus erythematosus in New Zealand: a population-based study. Rheumatology (Oxford).

[11] Campbell F, Biggs K, Aldiss SK (2016). Transition of care for adolescents from paediatric services to adult health services. Cochrane Database Syst Rev.

[12] Foster HE, Minden K, Clemente D (2017). EULAR/PReS standards and recommendations for the transitional care of young people with juvenile-onset rheumatic diseases. Ann Rheum Dis.

[13] (2025). GMC: Making decisions - professional standards. https://www.gmc-uk.org/professional-standards/the-professional-standards/0-18-years/making-decisions.

[14] Ruck Keene A, Kane NB, Kim SY, Owen GS (2019). Taking capacity seriously? Ten years of mental capacity disputes before England's Court of Protection. Int J Law Psychiatry.

[15] (2025). NHS: consent to treatment - children and young people. https://www.nhs.uk/tests-and-treatments/consent-to-treatment/children/.

